# Neural encoding of linguistic speech cues is unaffected by cognitive decline, but decreases with increasing hearing impairment

**DOI:** 10.1038/s41598-024-69602-1

**Published:** 2024-08-17

**Authors:** Elena Bolt, Nathalie Giroud

**Affiliations:** 1 Computational Neuroscience of Speech and Hearing, Department of Computational Linguistics, University of Zurich, 8050 Zurich, Switzerland; 2 International Max Planck Research School on the Life Course (IMPRS LIFE), University of Zurich, 8050 Zurich, Switzerland; 3 Language and Medicine Centre Zurich, Competence Centre of Medical Faculty and Faculty of Arts and Sciences, University of Zurich, 8050 Zurich, Switzerland

**Keywords:** Auditory speech processing, Linguistic speech processing, Cognitive decline, Natural continuous speech, Electroencephalography, Temporal response function, Neuroscience, Psychology, Biomarkers, Risk factors

## Abstract

The multivariate temporal response function (mTRF) is an effective tool for investigating the neural encoding of acoustic and complex linguistic features in natural continuous speech. In this study, we investigated how neural representations of speech features derived from natural stimuli are related to early signs of cognitive decline in older adults, taking into account the effects of hearing. Participants without ($n = 25$) and with ($n = 19$) early signs of cognitive decline listened to an audiobook while their electroencephalography responses were recorded. Using the mTRF framework, we modeled the relationship between speech input and neural response via different acoustic, segmented and linguistic encoding models and examined the response functions in terms of encoding accuracy, signal power, peak amplitudes and latencies. Our results showed no significant effect of cognitive decline or hearing ability on the neural encoding of acoustic and linguistic speech features. However, we found a significant interaction between hearing ability and the word-level segmentation model, suggesting that hearing impairment specifically affects encoding accuracy for this model, while other features were not affected by hearing ability. These results suggest that while speech processing markers remain unaffected by cognitive decline and hearing loss per se, neural encoding of word-level segmented speech features in older adults is affected by hearing loss but not by cognitive decline. This study emphasises the effectiveness of mTRF analysis in studying the neural encoding of speech and argues for an extension of research to investigate its clinical impact on hearing loss and cognition.

## Introduction

The increasing number of dementia patients in our ageing population underlines the urgent need for research that focuses on methods for early detection. Early stages of cognitive decline pose a major challenge for diagnosis and intervention, as early detection is central to effective treatment, making the identification of subtle changes prior to diagnosis particularly important^1^. Language performance, encompassing both comprehension and production, is intricately linked to cognitive functions and often deteriorates in dementia and other forms of cognitive decline^2–5^. The processing of language in the brain involves complex neural networks across various brain regions, which are vulnerable to early neuropathological changes, especially in conditions like Alzheimer’s disease (AD)^6^. Furthermore, the relationship between cognition and hearing is critically important^7^. Age-related hearing loss is closely linked with cognitive decline and is considered the most common modifiable risk factor for dementia^1^. A significant proportion of older adults with cognitive decline also experience hearing loss^8,9^. The manner in which the aging brain processes speech—particularly natural continuous speech that involves ongoing top-down and bottom-up processing^10^—through the auditory system may be key in the early detection of cognitive decline. Behavioral measures have indicated that individuals with early signs of cognitive decline show impaired auditory processing capabilities^11^. Neurophysiological studies have revealed that these early stages of decline are associated with significant changes in neural processing^12,13^. Specifically, altered encoding of syllable sounds at both cortical and subcortical levels has been observed in individuals with early cognitive decline, suggesting potential predictive value for such decline^12^. However, in our earlier work where natural speech was used instead of syllable sounds, we were unable to confirm these results^14^.

In our previous work, we used the temporal response functions (TRF) framework to focus on auditory speech encoding as reflected in both subcortical and cortical responses^15^. The TRF, a linearized stimulus-response model, delineates the relationship between speech input and neuronal response, as typically measured by electroencephalography (EEG). These models are particularly adept at quantifying the brain’s response to natural speech over extended listening periods and offer a broader temporal integration range than conventional evoked potentials. Natural speech, which has higher ecological validity than the syllable and click sounds typically used in conventional potentials, forms the basis of our study, increasing the applicability of the results in the real world. The ecological validity of natural speech is underscored by studies showing that cognitive factors, such as focal attention, significantly impact the cortical tracking of speech^16,17^. The concept of “neural tracking of speech” refers to the brain’s ability to follow the dynamic properties of the speech signal, which can be captured using TRF models. TRF models are critical for two reasons: they provide an encoding accuracy for the speech feature of interest and a time-delayed neural response function that enables neurophysiological interpretation^18^. Initially, our study focused on the encoding of an acoustic feature present in natural speech, specifically auditory nerve rates derived from speech wave rates—akin to a temporal envelope^14^. However, the flexibility of TRF models allows them to also fit a range of lexical (at the word-level) and sublexical (at the phoneme-level) speech features derived from the speech signal and its temporally aligned transcript, as has been done in several studies^19–22^. These models can be calculated using impulse vectors that code for, e.g., word or phoneme onsets, or scaled impulse vectors that reflect the surprisal value of a word or phoneme. Furthermore, a TRF model can simultaneously be modeled to multiple speech features, producing multivariate neural response functions—each corresponding to a different speech feature—known as a multivariate TRF (mTRF)^15^. The mTRF models extend beyond acoustic features to encompass linguistic features represented in natural speech. This framework has proven valuable in exploring differential acoustic and linguistic speech tracking in patients with post-stroke aphasia^22^ and demonstrating that linguistic representations diminish with increasing age^23^. Inspired by the opportunities offered by the mTRF framework, we have delved deeper into the investigation of linguistic processing.

**Figure 1:**
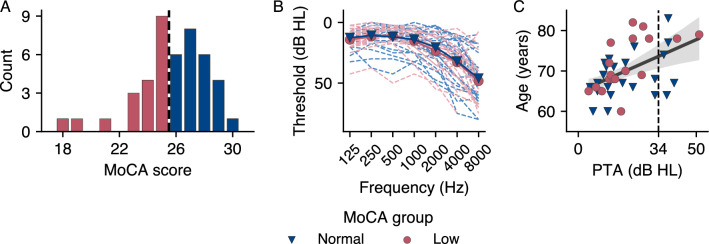
Montreal Cognitive Assessment (MoCA) scores, audiogram, and age as a function of four-frequency pure-tone average (PTA). (**A**) Distribution of MoCA scores. The dashed line indicates the cutoff score of 26. (**B**) Individual hearing thresholds for each frequency, averaged by ear and colored by MoCA group. PTA values calculated from the audiogram did not differ between groups. (**C**) Age as a function of PTA. The shaded area represents the 95% confidence interval. Age correlated with PTA across all participants. This plot is adapted from the original study^14^.

In this work, we drew on the dataset from our previous study^14^ to investigate the neural encoding of linguistic features in natural speech in older adults, focusing on participants with and without putative cognitive decline. Participants were categorized into two groups based on their scores from the Montreal Cognitive Assessment (MoCA)^24^: the normal MoCA group, showing no early signs of cognitive decline, and the low MoCA group, where participants scored below 26 points, the clinical threshold for mild cognitive impairment (MCI). The distribution of MoCA scores and the corresponding group allocation are shown in Fig. 1A.

**Figure 2:**
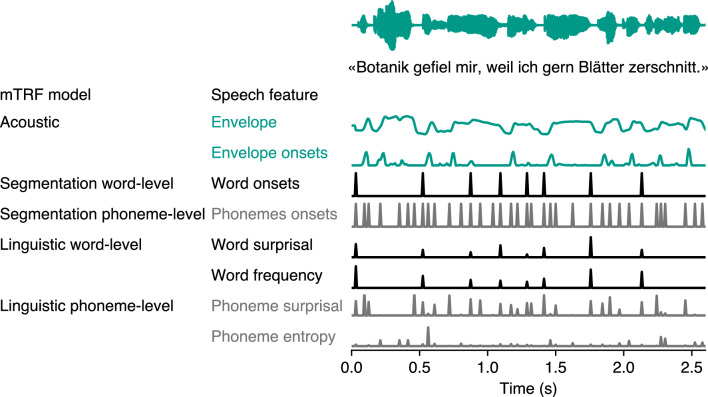
Overview over the multivariate temporal response function (mTRF) models with speech features. The features are demonstrated using an example sentence: “Botanik gefiel mir, weil ich gern Blätter zerschnitt.” (*I liked botany because I liked cutting up leaves*) from one of the audiobook segments used in the study. The acoustic model included the envelope and envelope onsets derived from the speech wave (teal colored). The word- and phoneme-level segmentation model included the word and phoneme onsets (black), the linguistic word-level model included the word surprisal and word frequency (black), and the linguistic phoneme-level model included the phoneme surprisal and phoneme entropy (gray), all derived from word- and phoneme-level time-aligned transcriptions. The graphical representation was inspired by the one created by Kries et al.^22^.

Drawing inspiration from the framework established by Kries et al.^22^, we conducted our analysis using five distinct mTRF models. These models covered a spectrum of linguistic features, from basic segmentation of words and phonemes to more complex analyses such as surprisal, frequency, and entropy of (sub)lexical items. Specifically, our models were designed as follows: the acoustic model incorporated the speech envelope and envelope onsets; the word- and phoneme-level segmentation models included the word and phoneme onsets; the linguistic word-level model included word surprisal and word frequency; and the linguistic phoneme-level model included phoneme surprisal and phoneme entropy (see Fig. 2). Given the collinearity between features originating from the same speech signal^25^, we regressed the features not of interest from the EEG signal before fitting the mTRF models to isolate the specific feature of interest for each model. Our exploratory approach aimed to determine whether the neural encoding of these linguistic features in natural speech varied between the two participant groups and to understand how these differences might be influenced by hearing loss.

Our analysis began with an evaluation of the encoding accuracy across the mTRF models to assess whether overall neural speech tracking performance was influenced by cognitive decline. We further explored the signal of the time-delayed neural response functions for the two speech features embedded in each mTRF model. This exploration aimed to investigate how the brain processes these features differently in participants with and without early signs of cognitive decline. Additionally, we integrated an assessment of hearing ability by quantifying the four-frequency pure tone average (PTA)^26^ to investigate potential interactions between auditory encoding, cognitive decline, and hearing ability. Individual hearing thresholds for each frequency, averaged by ear, are shown in Fig. 1B, and the interaction between age and PTA is shown in Fig. 1C. Considering the well-established link between cognitive decline and language processing^2–5^, we hypothesized that participants exhibiting early signs of cognitive decline would demonstrate altered neural encoding of linguistic features in natural speech, as evidenced by variations in encoding accuracy, compared to those without early signs of cognitive decline. We also anticipated that these differences would manifest in distinct response function signals and that hearing loss would modulate these effects, as seen in previous studies on neural speech processing (see, e.g.,^27–29^).

## Results

### Effect of MoCA group and PTA on encoding accuracy

To investigate how cognitive decline and hearing ability affect the encoding accuracy of the five different mTRF models—the acoustic, segmentation at the word- and phoneme-level, and linguistic at the word- and phoneme-level models, illustrated in Fig. 2—we implemented a linear mixed model (LMM) using orthogonal sum contrast coding for the categorical predictors. The results are detailed in Table 1.

**Figure 3:**
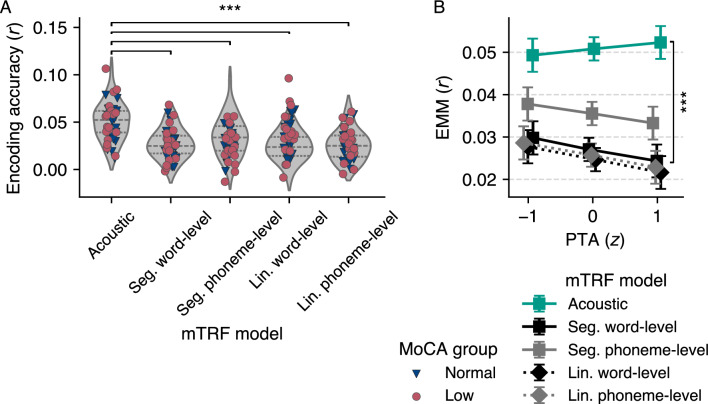
Encoding accuracy by multivariate temporal response function (mTRF) model and estimated marginal means (EMMs) of the interactions between four-frequency pure-tone average (PTA) and the mTRF models. (**A**) Encoding accuracies (Pearson’s *r*, averaged across all 32 electrodes) for each mTRF model. Violin plots show the distribution of the individual data points colored by Montreal Cognitive Assessment (MoCA) group, with the dashed line indicating the median and the dotted lines indicating the interquartile range. All mTRF models exhibited significantly lower encoding accuracies compared to the acoustic model. (**B**) EMMs of the interaction between PTA and the mTRF models. The acoustic model served as the reference level. The interaction was significant for the segmentation word-level model, with a greater difference in encoding accuracy observed as hearing ability decreased. The error bars represent the standard error of the mean. Significance levels are indicated as: $p < 0.001$ (***). Seg., segmentation; Lin., linguistic.

This statistical model accounted for individual differences and the nesting of the five encoding accuracies within participants, yielding the following insights: First, no significant main effect was observed for the MoCA group, indicating that encoding accuracies were comparable between participants with and without early signs of cognitive decline. Second, no significant main effect was detected for PTA, suggesting that hearing ability did not significantly influence encoding accuracy. Third, a significant main effect was found for the mTRF models: the acoustic model, serving as the reference level, exhibited higher encoding accuracy on average compared to the other models, Fig. 3A. Post-hoc tests confirmed that the encoding accuracies of all other models were significantly lower than that of the acoustic model ($p < 0.001$ for all comparisons, see Table S3). The interaction between MoCA group and PTA or between MoCA group and the mTRF models were not significant. A significant interaction was observed between PTA and the segmentation word-level model, whereas no other interactions between PTA and the mTRF models were significant, indicating that hearing impairment affects the encoding accuracy for this specific model. Additionally, no significant three-way interactions were found between MoCA group, PTA, and the mTRF models, indicating that the combined effect of cognitive decline and hearing impairment did not differentially affect the encoding accuracy across the different mTRF models.

To further explore the significant interaction between PTA and the segmentation word-level model, we conducted post-hoc tests. Specifically, we examined the estimated marginal means (EMMs) of the interaction at $-1$, 0, and $+1$ standard deviations of PTA, revealing the following: At PTA $z = -1$, the estimated difference in encoding accuracy between the acoustic and segmentation word model was 0.019 ($SE = 0.002$, $t(160) = 9.5$, $p < 0.001$). At PTA $z = 0$, the difference was 0.024 ($SE = 0.001$, $t(160) = 16.5$, $p < 0.001$). At PTA $z = 1$, the difference was 0.028 ($SE = 0.002$, $t(160) = 13.8$, $p < 0.001$). The EMMs for each mTRF model are visualized in Fig. 3B. These results indicate that, compared to the acoustic model, hearing impairment significantly affects encoding accuracy in the segmentation word-level model, with greater differences observed as hearing ability decreases.

Overall, our analysis suggests that while there is no overarching difference in encoding accuracy between participants with and without cognitive decline, hearing loss impacts encoding accuracy in the segmentation word-level model. Please note that the supplementary analysis treating MoCA as a continuous variable yielded similar conclusions (see Supplementary Analysis 1 and Table S1).

**Table 1:** Results of the linear mixed model (LMM) for the encoding accuracy of each multivariate temporal response function (mTRF) model.

Coefficient	$\beta$	$95\%$ CI		*df*	*t*	*p*	
		*LL*	*UL*				
Intercept	0.033	0.028	0.038	40	12.6	$< 0.001$	***
MoCA group (low)	$-0.002$	$-0.007$	0.004	40	$-0.6$	0.559	
PTA (*z*)	$-0.002$	$-0.007$	0.003	40	$-0.7$	0.475	
mTRF model (Seg. word-level)	0.018	0.016	0.020	160	19.9	$< 0.001$	***
mTRF model (Seg. phoneme-level)	$-0.006$	$-0.007$	$-0.004$	160	$-6.3$	$< 0.001$	***
mTRF model (Lin. word-level)	0.003	0.001	0.005	160	3.1	0.003	**
mTRF model (Lin. phoneme-level)	$-0.008$	$-0.010$	$-0.006$	160	$-8.9$	$< 0.001$	***
MoCA group × PTA	0.003	$-0.002$	0.008	40	1.3	0.205	
MoCA group × mTRF model (Seg. word-level)	$-2.3e-04$	$-0.002$	0.002	160	$-0.3$	0.800	
MoCA group × mTRF model (Seg. phoneme-level)	0.001	$-0.001$	0.003	160	1.3	0.211	
MoCA group × mTRF model (Lin. word-level)	$-0.001$	$-0.003$	0.001	160	$-1.4$	0.162	
MoCA group × mTRF model (Lin. phoneme-level)	0.001	$-0.001$	0.002	160	0.6	0.522	
PTA × mTRF model (Seg. word-level)	0.003	0.002	0.005	160	3.7	$< 0.001$	***
PTA × mTRF model (Seg. phoneme-level)	$-0.001$	$-0.003$	0.001	160	$-0.9$	0.357	
PTA × mTRF model (Lin. word-level)	$-3.8e-04$	$-0.002$	0.001	160	$-0.4$	0.679	
PTA × mTRF model (Lin. phoneme-level)	$-0.001$	$-0.003$	0.001	160	$-1.3$	0.205	
MoCA group × PTA × mTRF model (Seg. word-level)	0.001	$-0.001$	0.003	160	1.2	0.237	
MoCA group × PTA × mTRF model (Seg. phoneme-level)	$-0.001$	$-0.002$	0.001	160	$-0.6$	0.570	
MoCA group × PTA × mTRF model (Lin. word-level)	0.001	$-0.001$	0.002	160	0.7	0.464	
MoCA group × PTA× mTRF model (Lin. phoneme-level)	0.001	$-0.001$	0.002	160	0.7	0.500	

The LMM included Montreal Cognitive Assessment (MoCA) group, four-frequency pure-tone average (PTA), mTRF model and the interaction between MoCA group, PTA and mTRF model as fixed effects, and participant ID as a random effect. The reference levels for MoCA group and mTRF model were low and the acoustic model, respectively. Seg., segmentation; Lin., linguistic, CI, confidence interval; LL, lower limit; UL, upper limit; df, degrees of freedom. Orthogonal contrasts were used to test the interaction effects. Significance levels are indicated as: $p < 0.001$ (***), $p < 0.01$ (**), $p < 0.05$ (*).

### mTRF-model based effects of MoCA group and PTA on response function signal power

**Figure 4:**
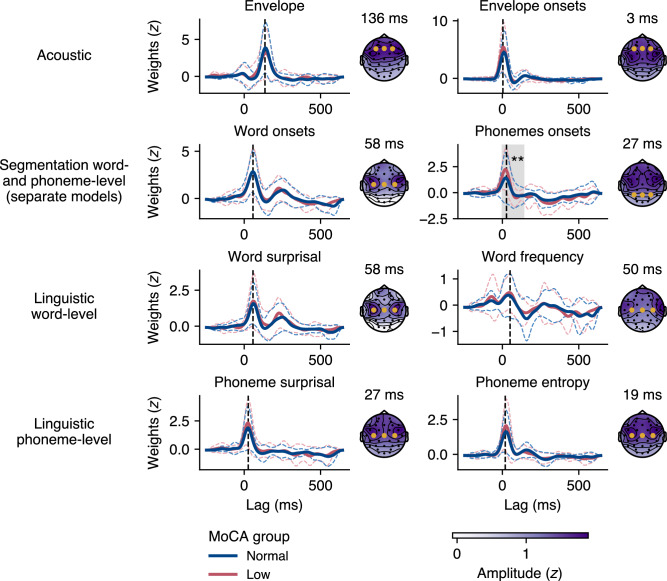
Mean weights of response functions for each speech feature in the multivariate temporal response function (mTRF) models by Montreal Cognitive Assessment (MoCA) group, with the low MoCA group indicating early signs of cognitive decline, and topographical maps. Response functions shown in the plot were globally *z*-transformed for visualization purposes. Topographical maps were derived from the largest average peak in the response functions, displaying the mean activity across all participants in a 50 ms window centered around the peak latency, as indicated by the vertical dashed line in the response functions. Displayed response functions are averaged from three key electrodes, marked in yellow on the topographical maps. The response functions showed distinct peaks that differed in amplitude and latency between the speech features. However, no significant differences in peak amplitudes or latencies were observed between the groups, with the exception of response peak latency in phoneme onsets in the parietal cluster during the early time window, as indicated by the shaded gray area. Significance levels are indicated as: $p < 0.01$ (**).

The response functions to each speech feature modeled in the mTRF models are depicted in Fig. 4. We evaluated the effects of cognitive decline on the signal power of these response functions by comparing the root mean square (RMS) values between participants with and without early signs of cognitive decline, using a LMM to account for individual differences and the nesting of RMS values within participants. Specifically, we ran the LMM separately for each mTRF model, with the RMS of the response functions to the different speech features nested within three electrode clusters (F, frontal; C, central; P, parietal) and participants. The results are summarized in Table 2.

Overall, the results largely paralleled those of the encoding accuracy analysis. First, no significant main effect was found for the MoCA group across any of the mTRF models, indicating that the signal power of the response functions did not differ between participants with and without cognitive decline. Second, PTA did not significantly affect the signal power for any of the mTRF models. Additionally, no significant interactions were found between MoCA group and PTA across any of the mTRF models. Third, the cluster variable was significant in all five LMMs, as reflected in the topographical maps of the response functions in Fig. 4. Parietal clusters exhibited lower RMS values compared to frontal clusters for all mTRF models, indicating that the signal power was more prominent in the frontal cluster. For the word- and phoneme-based models, central clusters showed significantly higher RMS values than frontal clusters, suggesting that in these higher-order linguistic models, the signal power was more distinct in central clusters. Finally, the speech feature variable also significantly influenced signal power, depending on the mTRF model. For the acoustic model, RMS for the envelope onsets was comparable to the envelope. In the segmentation model, signal power for phoneme onsets was significantly higher than for word onsets. In the word-based model, signal power for word frequency was significantly lower than for word surprisal. In the phoneme-based model, signal power for phoneme entropy was significantly lower than for phoneme onsets.

Taken together, these results suggest that the signal power of response functions to natural speech is not influenced by early signs of cognitive decline or hearing ability. However, the analysis indicates that the signal power of response functions to higher-order linguistic features is significantly influenced by electrode clusters and speech features. Also here it is noteworthy that the supplementary analysis treating MoCA as a continuous variable yielded similar conclusions (see Supplementary Analysis 2 and Table S2).

**Table 2:** Results of the linear mixed models (LMM) for the root mean square (RMS) of the temporal response function (TRF) patterns.

Coefficient	$\beta$	$95\%$ CI		*df*	*t*	*p*	
		*LL*	*UL*				
Acoustic model							
Intercept	0.001	0.001	0.001	49.7	11.9	$< 0.001$	***
MoCA group (low)	$6.0e-05$	$-2.1e-04$	$3.3e-04$	40.0	0.4	0.667	
PTA (*z*)	$9.4e-05$	$-8.2e-05$	$2.7e-04$	40.0	1.0	0.303	
Speech feature (envelope onsets)	$8.6e-06$	$-6.1e-05$	$7.8e-05$	745.0	0.2	0.809	
Cluster (C)	$-6.5e-05$	$-1.5e-04$	$2.0e-05$	745.0	$-1.5$	0.135	
Cluster (P)	$-0.001$	$-0.001$	$-4.7e-04$	745.0	$-12.8$	$< 0.001$	***
MoCA group × PTA	$-5.7e-05$	$-3.3e-04$	$2.2e-04$	40.0	$-0.4$	0.682	
Segmentation word-level model							
Intercept	0.001	0.001	0.001	43.6	11.1	$< 0.001$	***
MoCA group (low)	$4.3e-05$	$-2.2e-04$	$3.0e-04$	40.0	0.3	0.751	
PTA (*z*)	$-6.3e-06$	$-1.8e-04$	$1.6e-04$	40.0	$-0.1$	0.943	
Cluster (C)	$1.2e-04$	$5.7e-05$	$1.8e-04$	350.0	3.7	$< 0.001$	***
Cluster (P)	$-2.1e-04$	$-2.7e-04$	$-1.4e-04$	350.0	$-6.4$	$< 0.001$	***
MoCA group × PTA	$-1.1e-04$	$-3.8e-04$	$1.5e-04$	40.0	$-0.8$	0.404	
Segmentation phoneme-level model							
Intercept	0.001	0.001	0.001	43.2	9.6	$< 0.001$	***
MoCA group (low)	$2.2e-04$	$-1.6e-04$	0.001	40.0	1.1	0.269	
PTA (*z*)	$-4.1e-05$	$-2.9e-04$	$2.0e-04$	40.0	$-0.3$	0.746	
Cluster (C)	$4.1e-05$	$-4.4e-05$	$1.3e-04$	350.0	0.9	0.350	
Cluster (P)	$-4.9e-04$	$-0.001$	$-4.0e-04$	350.0	$-11.3$	$< 0.001$	***
MoCA group × PTA	$-3.2e-05$	$-4.1e-04$	$3.5e-04$	40.0	$-0.2$	0.870	
Linguistic word-level model							
Intercept	0.001	0.001	0.001	54.9	15.2	$< 0.001$	***
MoCA group (low)	$2.8e-05$	$-8.5e-05$	$1.4e-04$	40.0	0.5	0.627	
PTA (*z*)	$6.4e-06$	$-6.7e-05$	$8.0e-05$	40.0	0.2	0.865	
Speech feature (word frequency)	$-2.3e-04$	$-2.6e-04$	$-1.9e-04$	745.0	$-12.5$	$< 0.001$	***
Cluster (C)	$4.6e-05$	$2.0e-06$	$8.9e-05$	745.0	2.0	0.041	*
Cluster (P)	$-9.2e-05$	$-1.4e-04$	$-4.9e-05$	745.0	$-4.1$	$< 0.001$	***
MoCA group × PTA	$-7.4e-05$	$-1.9e-04$	$3.9e-05$	40.0	$-1.3$	0.208	
Linguistic phoneme-level model							
Intercept	0.001	0.001	0.001	47.1	11.3	$< 0.001$	***
MoCA group (low)	$7.5e-05$	$-8.0e-05$	$2.3e-04$	40.0	0.9	0.349	
PTA (*z*)	$-3.5e-05$	$-1.4e-04$	$6.5e-05$	40.0	$-0.7$	0.496	
Speech feature (phoneme entropy)	$-1.5e-04$	$-1.8e-04$	$-1.1e-04$	745.0	$-8.3$	$< 0.001$	***
Cluster (C)	$5.2e-05$	$9.8e-06$	$9.4e-05$	745.0	2.4	0.016	*
Cluster (P)	$-1.5e-04$	$-2.0e-04$	$-1.1e-04$	745.0	$-7.2$	$< 0.001$	***
MoCA group × PTA	$1.6e-05$	$-1.4e-04$	$1.7e-04$	40.0	0.2	0.844	

The models included Montreal Cognitive Assessment (MoCA) group, four-frequency pure-tone average (PTA), speech feature (in the acoustic and word- and phoneme-level linguistic models), electrode cluster (F, frontal; C, central; P, parietal), and the interaction between MoCA group and PTA as fixed effects, and participant ID as a random effect. The reference level for cluster was F. The reference levels for the mTRF models with two nested speech features were envelope (acoustic model), word surprisal (linguistic word-level model) and phoneme surprisal (linguistic phoneme-level model). CI, confidence interval; LL, lower limit; UL, upper limit, df, degrees of freedom. Significance levels are indicated as: $p < 0.001$ (***), $p < 0.01$ (**), $p < 0.05$ (*).

### Group comparisons of peak amplitudes and latencies

Our analysis of response functions to different speech features revealed distinct peaks that vary in amplitude and latency across responses, as illustrated in Fig. 4. We performed a detailed analysis of these peaks to investigate possible differences in peak amplitudes and latencies between participants with and without cognitive decline, in addition to the RMS analysis, which focuses primarily on signal power and neglects variations in peaks and their latencies. Our focus was on responses where at least $75\%$ of participants exhibited a peak (see summary in Table S4). The results of these comparisons, detailed in Table 3, predominantly showed no significant differences in peak amplitudes or latencies between the two groups during an earlier time window. However, an exception was noted for phoneme onsets in this time window. Specifically, participants with early signs of cognitive decline exhibited earlier peak latencies compared to those without cognitive decline in the parietal cluster (two-tailed Mann–Whitney U test, $U = 218.5$, Holm-Bonferroni corrected $p = 0.008$, $r = -0.41$). The descriptive statistics for all peak latencies are summarized in Table S5. On average, the peak latency for phoneme onsets was $43 \pm 40\,\hbox {ms}$ for the normal MoCA group and $17 \pm 12\,\hbox {ms}$ for the low MoCA group.

In the later time window, our analysis also revealed no significant differences in peak amplitudes or latencies across the different clusters for both groups. Overall, these findings suggest that response functions peaks of neural encoding of natural speech do not significantly differ between participants with and without early signs of cognitive decline, except for phoneme onsets in the early time window.

**Table 3:** Results of the Mann–Whitney U tests for peak amplitudes and latencies in early and late time windows. The table includes *U*-statistics, Holm-Bonferroni corrected *p*-values, effect sizes (Rank-Biserial correlation coefficient *r*), and the number of participants in each group. This analysis focused on peaks observed in more than $75\%$ of participants for each response and electrode cluster within each time window. Significance levels are indicated as: $p < 0.01$ (**).

Speech feature	Cluster	Amplitudes			Latencies				Participants	
		*U*	$p_{\text {corr}}$	*r*	*U*	$p_{\text {corr}}$		*r*	Normal	Low
Early window										
Envelope	F	289.0	1.000	$-0.28$	281.5	0.658		$-0.29$	25	18
	C	253.0	0.844	$-0.14$	264.5	0.973		$-0.12$	23	18
	P	255.0	1.000	$-0.16$	220.0	0.911		$-0.23$	22	16
Envelope onsets	F	176.0	0.568	$-0.17$	122.0	0.230		$-0.35$	21	15
Word onsets	F	182.0	1.000	$-0.19$	164.5	0.525		$-0.24$	22	17
	C	195.0	1.000	$-0.20$	180.0	0.475		$-0.23$	23	18
	P	137.0	1.000	$-0.10$	160.5	0.974		$-0.03$	17	17
Phoneme onsets	F	260.0	0.606	$-0.14$	228.0	0.821		$-0.20$	25	19
	C	247.0	0.885	$-0.12$	213.5	0.958		$-0.20$	24	18
	P	218.0	0.391	$-0.04$	82.5	0.008	**	$-0.44$	21	16
Word surprisal	F	125.0	1.000	$-0.17$	122.5	0.655		$-0.18$	18	15
	C	186.0	1.000	$-0.17$	176.0	0.551		$-0.19$	22	18
	P	176.0	1.000	$-0.04$	125.5	0.255		$-0.20$	19	16
Phoneme surprisal	F	212.0	0.298	$-0.12$	151.5	0.467		$-0.29$	21	15
	C	230.0	0.393	$-0.12$	160.5	0.502		$-0.31$	22	15
Phoneme entropy	F	212.0	0.893	$-0.12$	179.0	0.939		$-0.21$	21	16
	C	196.0	0.808	$-0.10$	155.0	0.693		$-0.23$	21	16
	P	178.0	0.526	$-0.17$	90.5	0.031		$-0.45$	21	15
Late window										
Word onsets	F	221.0	0.960	$-0.13$	179.5	0.330		$-0.22$	22	17
	C	162.0	1.000	$-0.21$	152.0	0.633		$-0.24$	20	16
	P	233.0	1.000	$-0.30$	208.0	0.735		$-0.37$	22	15
Word surprisal	F	267.0	1.000	$-0.07$	193.0	0.398		$-0.23$	22	18
	C	131.0	0.901	$-0.15$	138.0	0.928		$-0.12$	18	15
	P	190.0	1.000	$-0.11$	206.5	0.419		$-0.07$	19	15
Word frequency	F	187.0	0.829	$-0.23$	164.5	0.403		$-0.29$	20	15
	P	150.0	0.610	$-0.28$	141.5	0.202		$-0.30$	18	15

## Discussion

The primary aim of our study was to explore how older adults, both with and without early signs of cognitive decline, encode acoustic, segmentation, and linguistic cues in natural continuous speech, while also considering the impact of hearing ability. We used the mTRF framework to analyze neural responses to various acoustic, lexical, and sublexical features in an audiobook’s speech stream. This analysis serves as a proxy for understanding how the brain processes these features, using EEG data from our previous study^14^. Our analysis focused on encoding accuracy, signal power, and response peak and latencies across five models: acoustic, segmentation at the word- and phoneme-level, and linguistic at the word- and phoneme-level.

Contrary to our hypothesis, our findings revealed no significant impact of cognitive decline on neural encoding of speech as measured by our metrics, despite existing literature suggesting a link between cognitive decline and deteriorating language performance^2–5^. Similarly, we found no significant main effect of hearing ability on neural encoding of speech features, though hearing loss—a common comorbidity in cognitively declining older adults—typically alters speech processing^8,9,27,28,30^. This is surprising, especially considering the common pool model of cognitive processing resources^31^, which suggests that cognitive decline and hearing loss may compete for limited cognitive and perceptual resources, thereby reducing neural encoding of speech features in affected individuals. This theory suggests that if participants with advanced cognitive decline or hearing loss were included, differences in neural encoding might be more pronounced, particularly in tasks requiring more cognitive resources, such as speech-in-noise perception or in individuals with diagnosed MCI or AD. For a related discussion on natural speech tracking with and without visual enhancement, which treats resources differently, see Frei et al.^32^. In our data, we did not observe such an association between cognitive decline and neural encoding, suggesting that early cognitive decline did not significantly affect the neural processing of speech in our participants.

Cognitive factors are known to influence neural tracking of speech. Studies have shown that neural speech tracking, particularly the phase-locking of the neural response to the amplitude envelope, is crucial for successful speech comprehension^33–35^. Studies also demonstrated that neural speech tracking underlies successful speech comprehension^29^, with a positive relationship observed between neural tracking and speech comprehension in older adults with both normal hearing and hearing impairment^27,36^. Furthermore, research has indicated that the older brain recruits additional higher-level auditory regions during the early stages of speech processing to maintain speech comprehension^19,37^. Thus, both cognitive decline and hearing loss are known to affect neural encoding of speech, with potential implications for speech comprehension. The lack of a significant effect of cognitive decline on neural encoding in our study may be due to the relatively early stage of cognitive decline in our participants, as indicated by the MoCA scores, see also the limitations discussed below.

What we did observe was a significant interaction between hearing ability and the segmentation word-level model, indicating that increasing hearing impairment led to a decrease in the brain’s ability to track word segmentation in natural speech. This is noteworthy since recognizing words in continuous speech is a complex task requiring substantial cognitive resources, which can become even more challenging in the presence of hearing loss^38,39^. It is intriguing that we did not see these interactions in the higher-order linguistic models, nor did we observe a significant main effect of PTA on neural encoding accuracy, which is commonly reported in other studies^27,28,30^. We also found that while investigating the word-level segmentation mTRF model at the response signal level, hearing ability did not affect the response signal power, contrasting with the encoding accuracy results.

Another noteworthy result was the significantly earlier peak latency for phoneme onsets in participants with early signs of cognitive decline compared to those without, observed in a parietal cluster during the early time window. Given that phoneme processing is more demanding than word processing^40^, it is possible that the earlier latencies reflect a compensatory mechanism or a heightened sensitivity to processing demands in individuals with cognitive decline. The emergence of this result in a parietal cluster is also consistent with the literature, as the parietal cortex is known to be involved in phonological processing^41^. Additionally, a previous study demonstrated a decrease in encoding accuracy in linguistic neural tracking with age, particularly in a comparable parietal region^21^.  This could suggest that cognitive decline impacts the timing of more demanding phonological processes, leading to these earlier neural responses.

Overall, our study suggests that while there is no overarching difference in speech encoding between participants with and without cognitive decline, hearing loss specifically impacts word segmentation in speech processing.

### Implications for the study of cognitive decline and hearing loss

Drawing on our previous research^14^, we anticipated no significant differences in the acoustic encoding of speech in this dataset. However, we did expect to observe differences in the encoding of linguistic features, particularly in participants with early signs of cognitive decline, which we did not find. Our interest was in exploring potential variations in neural encoding of linguistic features, given the established link between language performance and cognitive functions, which may alter under cognitive decline. Previous results where we applied machine learning methods to voice parameters extracted from a semi-spontaneous speech task recorded with the same participants, we were able to classify the low MoCA group with a cross-validated accuracy of $71\%$^42^. In our current study, in contrast, we found no significant differences in neural encoding of linguistic features between participants with and without early signs of cognitive decline. The neural responses appeared relatively homogeneous across participants, regardless of their cognitive status, suggesting that neural encoding of speech in natural continuous settings may not effectively indicate early cognitive decline in older adults. Regarding hearing ability, our findings diverged from existing studies, where hearing loss often correlated with enhanced cortical speech tracking measures in older adults^27,28,30^. The relatively good hearing ability in our sample, with only seven individuals exhibiting a PTA exceeding $34\,\hbox {dB HL}$, which can be considered as moderate hearing loss^43^, might explain the lack of a significant association between hearing ability and neural encoding measures. We hypothesize that a sample with a higher prevalence of hearing loss might have yielded different results. The lack of a significant main effect of cognitive decline on neural encoding suggests that early cognitive decline might not uniformly disrupt neural processing of speech at the acoustic and linguistic levels. In addition, the specific impact of hearing loss on word-level segmentation underscores the importance of auditory cues in successful word recognition and highlights the additional cognitive load imposed by hearing impairment^39^.

### Dual role of encoding accuracies in mTRF modeling

Our study found differences in encoding accuracies across the five mTRF models: acoustic, segmentation at the word- and phoneme-level, and linguistic at the word- and phoneme-level. The acoustic model consistently showed higher encoding accuracy compared to the other models. In contrast, the segmentation models, particularly at the phoneme-level, exhibited lower encoding accuracies. There is a high correlation between speech features like the ones we used^25^, which we accounted for as detailed in the Methods section. Specifically, the envelope of the speech signal, which is the basis for the acoustic model, contains information about the speech rate, rhythm, and prosody^44^, while the segmentation and linguistic models are more isolated to boundaries and feature-engineered linguistic cues. Furthermore, the speech envelope is a continuous signal, while the segmentation and linguistic models are based on discrete features and are directly derived from the speech wave heard by participants. This distinction likely accounts for the differences in encoding accuracies, as the acoustic signal contains more direct, continuous auditory information compared to the derived linguistic features.

Initially, encoding accuracy in mTRF models was introduced as a measure to assess the quality of the model, involving statistical testing^18^. Specifically, one approach is to establish a null distribution using a permutation test procedure to define a null distribution against which the accuracy score is tested^18^. Similarly, some researchers establish a noise floor by phase scrambling the target regressors and then comparing the encoding accuracies from the original data to the noise floor^28,45^. However, other researchers have started using encoding accuracy as a measure of neural tracking, reflecting how well the brain follows specific features of the speech signal^22,25^, using it as a proxy for how well the speech representation is reflected in the EEG signal. The review by Gillis et al.^25^ discusses the use of encoding models as a diagnostic tool to assess the auditory pathway, highlighting this dual role. This dual role of encoding accuracy is a crucial methodological consideration when using the mTRF framework to study neural speech processing. In our data, we found that encoding accuracy is highly correlated with the signal power of the response signal (see Figure S2 for the correlation between encoding accuracy and signal power). This observation aligns with the notion that both the quality of the model and the neural tracking of speech can be inferred from the encoding accuracy.

The methods used to establish encoding accuracies also vary. Some scholars use a nested cross-validation approach to estimate encoding accuracies, as we did in the current manuscript, where the test set is also rotated (e.g.,^28,46^), while others use a leave-one-trial-out cross-validation approach (e.g.,^47^), as recommended in the sample procedure outlined by Crosse et al.^18^ and which we did in our previous study^14^. Depending on this procedure, the encoding accuracies can vary, which is an important consideration when comparing results across studies. It is noteworthy that there are no established benchmark values for expected encoding accuracies when comparing different acoustic, linguistic, and segmentation models.

Given the strengths of the mTRF framework, which provides both a signal response that allows for neurophysiological interpretation^15^ and a general encoding accuracy^25^, it is important to have clear research recommendations. Future studies should aim to delineate when to interpret response signals in terms of their power and shape, including time lags, and when it is more appropriate to focus on encoding accuracies. Establishing guidelines and normative values for these interpretations would enhance the reliability and consistency of findings in neural speech processing research.

### Limitations of the study and future directions

*Neuropsychological assessment*: A major limitation of our study was that we relied on the MoCA as a proxy for detecting putative MCI, i.e., early signs of cognitive decline. We chose this approach for two main reasons: First, ideally, we would have conducted this study with patients diagnosed with MCI or AD, but it proved difficult to recruit participants with a clear neurocognitive profile—a common issue in early cognitive decline studies. Therefore, we used the MoCA, a screening instrument for MCI, to provide a rough classification of our participants. Second, we preferred using the MoCA over a neurocognitive test battery, as the MoCA was specifically developed for screening evaluations. Although the MoCA is suitable for initial screening, it may not be sensitive enough to detect subtle cognitive changes in our participants. Furthermore, the stage of cognitive decline detected with the MoCA may be too early to significantly affect neural speech encoding. Therefore, in replicating the study, we would rely on more comprehensive neuropsychological assessments that allow for a more accurate classification of cognitive status and a deeper understanding of the relationship between cognitive decline and neural speech encoding. In addition to the MoCA, it would have been beneficial to administer a more comprehensive, age-appropriate IQ test. An example for our case would be the LPS $50+$, which assesses cognitive status and intellectual profiles in individuals aged 50–90 years and aids in diagnosing brain function disorders (e.g., early detection of degenerative diseases)^48^. This would allow us to relate the MoCA cut-off scores to the IQ test results, providing a more comprehensive picture of the cognitive status of our participants. We suggest that future studies incorporate such assessments to more accurately describe these relationships.

*Stimulus complexity and cognitive demand*: The complexity of the auditory stimulus in our study, involving the presentation of an audiobook in silence, might not have been sufficiently challenging to reveal differences in neural encoding between participants with and without early signs of cognitive decline. Prior research from our lab has shown that neural tracking of speech in older adults is significantly affected by cognitive load, particularly in speech-in-noise conditions^29^. Thus, embedding natural speech stimuli in more demanding listening conditions could provide a more robust assessment of the impacts of cognitive decline on speech processing, representing a promising direction for future research.

*Methodological considerations in neural encoding*: The methodology we used to investigate neural speech processing, particularly the use of the mTRF framework, faced limitations worth noting. The interdependence of the speech features used in our study posed a challenge in accurately isolating the effects of individual features on neural encoding. We chose to address this by regressing out the shared variance between features, which is one of several approaches, see review by Gillis et al.^25^. While this method helps mitigate some confounding effects, it may still have influenced the outcomes of our models. Furthermore, the linguistic models used—specifically, the word- and phoneme-based models—were constrained by the available features. Unlike previous studies that used 5-gram language models to estimate word surprisal, such as, e.g., Kries et al.^22^ or Gillis et al.^49^, we adopted a different approach due to our resource constraints. We used German BERT^59^, a pretrained large language model, to estimate word surprisal, potentially yielding different estimates compared to those derived from 5-gram language models. For phoneme surprisal, we designed a custom phonetic lexicon based on the Montreal Forced Aligner’s (MFA) pronunciation dictionary, and used the DeReKoGram frequency dataset^50^, which is an important source for behavioral or neurophysiological studies that require a large-scale corpus, for word frequencies. Additionally, see Weissbart et al.^20^ for a customized approach to *n*-gram models for estimating word surprisal. While our method allowed for bespoke estimations, it may have introduced inaccuracies in word- and phoneme-based speech feature calculations, potentially affecting the results of our mTRF models. Future research should strive for a unified methodological approach in constructing auditory encoding models. This would facilitate direct comparisons between studies and ensure more consistent estimations of linguistic surprisals, enhancing the reliability and interpretability of findings in cognitive neuroscience.

### Conclusion and outlook

Our study revealed a significant interaction between hearing ability and the segmentation word-level model. Participants with reduced hearing ability showed lower encoding accuracy for the word segmentation model, suggesting that hearing impairment specifically affects the neural encoding of word boundaries in natural speech. This finding underscores the importance of auditory cues in successful word recognition and highlights the additional cognitive load imposed by hearing impairment. While we did not observe significant differences in the neural encoding of linguistic features between older adults with and without early signs of cognitive decline, our findings emphasize the need to consider the interplay between cognitive and auditory functions, particularly in the context of hearing loss. Future studies should incorporate more comprehensive neuropsychological assessments and introduce more challenging listening conditions to better understand these relationships. These enhancements would improve our understanding of the relationship between cognitive decline, hearing impairment, and neural encoding of speech in older adults. Additionally, recruiting individuals with more severe cognitive decline in future research could provide insights into the effects at different stages of cognitive impairment. Our work contributes to the expanding literature on the intersection of cognitive decline and hearing loss, highlighting the complex interactions between auditory and cognitive functions in older adulthood.

## Methods

### Participants and cognitive grouping

This reanalysis included 44 native Swiss-German speakers from the first study^14^. Participants were monolingual up to the age of seven, right-handed and retired. Exclusion criteria included a professional music career, dyslexia, significant neurological disorders, severe or asymmetrical hearing loss and the use of hearing aids. The study was conducted in accordance with the Declaration of Helsinki and approved by the Ethics Committee of the Canton of Zurich (BASEC No. 2019-01400), with all research methods conducted in adherence to the relevant guidelines and regulations. The sessions were conducted at the Linguistic Research Infrastructure (LiRI, liri.uzh.ch). Written informed consent was obtained from all participants and they received compensation for their participation.

Participants were divided into groups based on the MoCA^12,24^. MoCA scores range from 0 to 30, with a cutoff of 26 for normal cognitive function. Participants who scored 26 or more were assigned to the normal MoCA group, participants below 26 to the low MoCA group. The normal MoCA group included 25 participants (14 women) with an average age of $68.6 \pm 5.3$ years (range: 60–83 years). The low MoCA group included 19 participants (12 women) with a mean age of $71.7 \pm 6.3$ (range: 60–82 years). The age differences between the groups were not statistically significant (two-tailed Mann–Whitney U test: $U =$311.5, $p = .081$, $r = 0.31$). The distribution of MoCA scores is shown in Fig. 1A.

### Audiometry

We measured the hearing thresholds for pure tones at frequencies of 125, 250, 500, 1000, 2000, 4000, and $8000\,\hbox {Hz}$ in both ears, using the Affinity Compact audiometer (Interacoustics, Middelfart, Denmark), equipped with a DD450 circumaural headset (Radioear, New Eagle, PA, USA). Individual frequency thresholds are shown in Fig. 1B. Overall hearing ability was determined using the four-frequency PTA, the average of the thresholds at 500, 1000, 2000 and $4000\,\hbox {Hz}$^26^. The average interaural difference was $4.5 \pm 4.4\,\hbox {dB HL}$ (range: 0–$17.5\,\hbox {dB HL}$), indicating symmetrical hearing ability. The PTA, averaged over both ears, was $19.6 \pm 11.8\,\hbox {dB HL}$ for the normal MoCA group and $20.4 \pm 11.5\,\hbox {dB HL}$ for the low MoCA group. Most participants showed no to mild hearing impairment, while seven showed moderate impairment ($\hbox {PTA} > 34\,\hbox {dB HL}$): two in the low MoCA group and five in the normal MoCA group. Furthermore, PTA was correlated with age ($\rho = 0.5$, $p = .0003$, Fisher’s $z = 0.57$, $n = 44$), see Fig. 1C. No significant group differences in hearing ability were found (two-tailed Mann–Whitney U test: U = 262.5, p = .561, r = 0.11). However, as hearing ability is relevant and has an influence on auditory encoding^21,27,28^, we included PTA as a control variable in the analyses.

### EEG recording

We recorded the EEG using the Biosemi ActiveTwo system (Biosemi, Amsterdam, The Netherlands) with 32 electrodes (10–20 system), four external electrodes and a sampling rate of $16.384\,\hbox {kHz}$. We positioned two external electrodes above and below the right eye to measure the electrooculogram (EOG), and two on the left and right mastoids. Participants sat in a soundproof, electromagnetically shielded booth during the recording. During the listening tasks, we instructed participants to focus on a fixation cross displayed on a screen and to minimize movement, especially when the cross was visible. Throughout the experiment, we monitored and maintained electrode offsets below $20\,\upmu \hbox {V}$.

### Audiobook

Participants listened to 25 segments from the German audiobook version of Sylvia Plath’s novel, “The Bell Jar,” read by a professional female speaker^36^. Segments lasted on average $45.7 \pm 2.7\,\hbox {s}$, totalling a listening time of approximately $20\,\hbox {min}$. Silent gaps were limited to $500\,\hbox {ms}$ and the average speech wave intensity was scaled to $70\,\hbox {dB}$ SPL. Each segment commenced with a short silence of approximately $467\,\hbox {ms}$, which we retained to introduce stimulus onset jitter. We calibrated the sound level such that segments were consistently played at $70\,\hbox {dB}$ peSPL. Audiobook segments were presented bilaterally through electromagnetically shielded insert ER3 earphones (Etymotic Research, Elk Grove Village, IL, USA)

### Signal processing

We performed all data processing in Python 3.11.6 and used the MNE-Python package^51^ for all signal preprocessing steps. Unless otherwise specified, all filters applied to the EEG and speech wave signals were non-causal Infinite Impulse Response (IIR) Butterworth filters with an effective order twice the specified filter order, which was always set to 3. Anti-alias filters were consistently applied at $\frac{1}{3}$ of the target rate.

#### EEG

First, we removed bad electrodes—on average $1.5 \pm 1.6$ electrodes per participant—and then referenced the EEG signals to the mean of the two mastoid channels. For six participants with at least one noisy mastoid channels, we used cap electrodes T7 and T8 as reference. We then segmented the continuous EEG from $-4$ to $54\,\hbox {s}$ relative to audiobook onset and downsampled the epochs to $512\,\hbox {Hz}$ after applying an anti-alias filter at $170.7\,\hbox {Hz}$. We used Independent Component Analysis (ICA) to remove artifacts from the EEG signals. We created a copy of the epoch instance for ICA and filtered the epoch copy with a high-pass filter at $1\,\hbox {Hz}$ (zero-phase, non-causal Hamming window Finite Impulse Response (FIR) filter, transition bandwidth: $1\,\hbox {Hz}$, filter length: 1691 samples), a process reported to facilitate ICA decomposition^52^. We performed ICA using the Picard algorithm^53^ with 1000 iterations, aiming to obtain $99.9\%$ of the variance of the signal. Furthermore, we improved the ICA performance by using five iterations within the FastICA algorithm^54^. After ICA fitting, the components associated with eye-related artifacts were automatically labeled using the external EOG electrodes as references, and the components associated with muscle activity or singular artifacts were manually labeled based on topography, temporal occurrence, and frequency spectrum. On average, we excluded $2.5 \pm 1.1$ components per participant. We zeroed out the components in the original epoch instance and then performed electrode interpolation for the epochs. The EEG was then downsampled to $128\,\hbox {Hz}$ after applying an anti-alias filter at $42.7\,\hbox {Hz}$. Finally, we band-pass filtered the EEG between 0.5 and $25\,\hbox {Hz}$. To facilitate matrix storage, the epochs were cut to 0 to $45\,\hbox {s}$ relative to audiobook onset.

#### Speech wave

The speech waves from each segment were processed to extract acoustic features. We first downsampled the speech waves to $15\,\hbox {kHz}$ using an anti-alias filter at $5000\,\hbox {Hz}$. Speech waves were then passed through a Gammatone filterbank^55^ with 28 channels with center frequencies from 50 to $5000\,\hbox {Hz}$ spaced equally on the equivalent rectangular bandwidth (ERB) scale. Each Gammatone frequency channel output was half-wave rectified and raised to the power of 0.3, before we avaraged the filter outputs across channels to obtain a univariate temporal envelope. In line with the EEG preprocessing, we downsampled the envelopes to $128\,\hbox {Hz}$ after applying an anti-alias filter at $42.7\,\hbox {Hz}$, and eventually band-pass filtered them between 0.5 and $25\,\hbox {Hz}$. We then truncated the envelopes to a uniform length of $45\,\hbox {s}$ or padded them with zeros (depending on the segment length).

### Acoustic and linguistic speech features extraction

Our goal was to model neural responses not only to acoustic features of the speech signal, but also to a range of lexical (word-based) and sublexical (phoneme-based) representations in the signal. To this end, we generated a range of time-aligned linguistic speech representations as impulse features from the audiobook transcription. Drawing inspiration from Kries et al.^22^, we constructed five models, each with distinct speech features, to quantify the tracking of different levels of linguistic information in the speech signal. These models included (1) an acoustic model, (2) a segmentation model at the word-level, (3) a segmentation model at the phoneme-level, (4) a linguistic model at the word-level, and (5) a linguistic model at the phoneme-level. We generated the speech features for each segmentation and linguistic mTRF model as vectorized time series of zeros, with a sampling rate of $128\,\hbox {Hz}$ and a length of $45\,\hbox {s}$, corresponding to the length and rate of the EEG epochs. Features were modeled as impulses at word and phoneme onsets, respectively. An example of the speech features is shown in Fig. 2.

#### Acoustic features

The speech feature pair for the acoustic model were the *envelope* and the *envelope onsets*, both of which were extracted from the speech wave. The extraction of the envelope is described in the previous section. Since the brain is sensitive to contrast and changes and information is often encoded in acoustic onsets in particular^56^, the model also included the envelope onsets. We constructed the envelope onsets as the half-wave rectified derivative of the envelope.

#### Segmentation features at word- and phoneme-level

Speech features of segmentation consisted of *word onsets* and *phoneme onsets*. For extracting the onsets, we determined their boundaries using the MFA (version 2.2.14)^57^. We created a transcript in Praat and then used the pre-trained German MFA acoustic model and the German MFA pronunciation dictionary^58^ to first, create a phonetic transcription of the audio file and second, to determine the timing of word and phoneme boundaries for each segment. The accuracy of word and phoneme boundaries, along with their time-aligned transcriptions, was manually verified and corrected as necessary. Word and phoneme onsets timepoints were then extracted from the MFA output and modeled as impulses of the value one in the vectorized time series of zeros.

#### Linguistic word-level features

The linguistic word-level model included *word surprisal* and *word frequency* as speech features. Word surprisal is an approximation of how unexpected a word is in a given context. We used the pre-trained German BERT model^59^ for this calculation. BERT, short for Bidirectional Encoder Representations from Transformers, is a state-of-the-art language model designed for contextual language analysis. We adapted BERT to simulate unidirectional (left-to-right) context processing, reflecting natural listening comprehension. For each word in the audiobook segments, we created a sequence with the target word masked. The model then predicted the probability of the masked word based on its preceding context. We calculated word surprisal as the negative logarithm of the probability predicted by BERT for each segment.

Word frequency describes the frequency of a word out of context. We used DeReKoGram, a novel frequency dataset for 1-, 2- and 3-grams from the German Reference Corpus^50^, using the unigram frequencies to calculate the frequency of each word^20^. Relative unigram frequencies can be viewed as an estimate of the unconditional probability of occurrence of a word. First, we determined the relative frequency of each word in the audiobook segment-based transcript, and then we used the negative logarithm of this value as the word frequency. This procedure results in words with a high frequency producing a low value and vice versa^22,49^.

#### Linguistic phoneme-level features

The speech features in the linguistic phoneme-level model were *phoneme surprisal* and *phoneme entropy*. Phoneme surprisal reflects how surprising a phoneme is given the preceding phonemes. We calculated phoneme surprisal as the negative logarithm of the phoneme probability given an activated cohort of phonemes, in line with prior work^22,49,56^. We generated a phonetic lexicon with lexical statistics by combining pronunciations from the MFA pronunciation dictionary and word frequency derived as absolute unigram frequencies from the DeReKoGram dataset, in which missing pronunciations were manually added, and words occurring in the stimuli but missing from DeReKoGram were assigned a frequency of one^56^. We then used the lexicon to calculate the probability of each phoneme given the preceding phonemes in the activated cohort and thus derived surprisal values for each phoneme in the audiobook segments.

Phoneme entropy is an indicator for the degree of competition between words congruent with the current phonemic input^49,56^. At the beginning of a word utterance, a large number of potential words form the activated cohort, leading to a high level of competition. This competition decreases as the utterance progresses and the cohort becomes smaller. We calculated the phoneme entropy using the Shannon entropy formula applied to the words within the activated cohort. Specifically, the initial phoneme of each word included all words in the active cohort. We used the same phonetic lexicon as for phoneme surprisal to calculate the phoneme entropy.

### Regressing out speech features not of interest

Following the methodology of Kries et al.^22^, we addressed the problem of collinearity between features before fitting the mTRF models. Given the interdependence of features—e.g., envelope onsets include information about word onsets and word onsets reflect phoneme onsets (and vice versa)—it was crucial to isolate the specific feature of interest for each model^25^. Before fitting the mTRF models, we regressed out features not of interest for the current model from the EEG signal. The reason for this is the collinearity between the features, for example, the envelope onsets contain information about word onsets, and word onsets contain information about phoneme onsets, but also vice versa, sublexical features can reveal information about lexical features^25^. We therefore regressed out the features of no interest from the EEG signal and then used the EEG residuals for the mTRF models. We did this using a linear regression model with the EEG signal as the dependent variable and the non-interesting features as independent variables. We then used the residuals, which were the difference between the EEG signal and the predicted EEG by the linear model, as target for the mTRF models. For the acoustic models, we regressed out the features for word- and phoneme-level segmentation and linguistic models. Regarding the segmentation models at both the word and phoneme levels, we regressed out the features for the acoustic model and the word- and phoneme-level linguistic models, but not for each other^22^. When addressing the word-level linguistic models, we regressed out the regressors for the acoustic, segmentation, and phoneme-level linguistic models. Similarly, in the phoneme-level linguistic models, we regressed out the regressors for the acoustic, segmentation, and word-level linguistic models.

### mTRF modeling

We quantified the cortical encoding of the speech features using mTRF models computed with the Eelbrain toolbox^60^. Eelbrain applies the Boosting algorithm to fit the mTRF models while mitigating the overfitting that is present in correlated features as in our data^61,62^. Boosting is a coordinate descent algorithm that iteratively updates a sparse multi-temporal resolution filter (the mTRF model) by changing a single filter weight at a time based on training data, as opposed to the uniform filter weight adjustment of ridge regression, which is also a conventional method in TRF modeling^15^. After each update of the weights, the model is evaluated against validation data and training stops when error reduction ceases, preventing overfitting and ensuring that irrelevant filter weights remain at zero, thereby increasing the parsimony of the model. A detailed explanation of the Boosting algorithm in the Eelbrain toolbox can be found in Brodbeck et al.^60^.

For each participant, we fitted five mTRF models. To prevent overfitting the models to the onset effects of the speech wave, we truncated the first second of each speech feature and EEG time series before fitting the mTRF models^18^. Upon fitting the models using the Boosting algorithm, we adjusted the basis function in Eelbrain to $1 \times 10^{-3}$ and normalized the feature-target pairs by *z*-transformation, setting the scale_data parameter to True. The models were fitted for time lags ranging from $-200$ to 600 ms. We used a six-fold cross-validation approach in which the feature-target pairs based on 25 audiobook segments were systematically rotated through the training, validation, and testing phases. Each segment was used four times for training, once for validation, and once for testing across all folds. The models were calibrated using the training segments, optimized using the validation segments, and evaluated using the test segments, with encoding accuracy assessed using the Pearson correlation coefficient between the observed EEG residuals *y* and the predicted EEG residuals $\hat{y}$. The mean correlation coefficient across all validation folds was calculated for each electrode, resulting in a single metric for encoding accuracy per electrode. Consequently, each mTRF model yielded one correlation coefficient per electrode and two response functions (for acoustic and linguistic models) or one response function (for segmentation models) per speech feature. Note that only the acoustic and linguistic models are actual mTRF models, while the segmentation models are not multivariate, thus TRF models only, but for the sake of consistency, we refer to all models as mTRF models. We averaged the encoding accuracies across all electrodes to obtain a single, comprehensive value of encoding accuray, and stored it together with the response functions for further analysis.

### Spatial and temporal clustering of response functions

#### Electrode clusters

We selected nine a priori midline electrodes for further analysis: F3, Fz, F4, C3, Cz, C4, P3, Pz, and P4, which were categorized into three cluster regions: frontal (F), central (C), and parietal (P). This approach allows to include the activity of individual electrodes in the analysis and at the same time to perform hierarchical modeling at the cluster level. The electrodes were selected based on their proximity to the auditory cortex and their relevance to neural speech processing, while covering a broad area of the scalp without making prior assumptions about the exact location of the neural generators of the response functions.

#### Temporal clusters

Previous studies have shown that peaks in the response functions occur in different time windows depending on the speech feature of interest^21,22^. To determine the time windows, we took a data-driven approach as follows: First, for each participant and each of the nine a priori selected electrodes, we identified the two largest peaks in each response function. We extracted peaks within time lags of $-50$ to 600 ms, omitting most of the negative lags but accounting for potential peaks occurring close to time lag zero. We used the find_peaks function from SciPy to identify the peaks, setting the prominence parameter to 0.5, meaning that the peak must be at least 0.5 times higher than the surrounding data points. Finally, we excluded peaks with latencies below 0 ms, since neurophysiological responses are not expected to occur before the stimulus. Second, using the latencies of the two largest peaks across all electrodes, we performed K-Means clustering to group the time lags into two clusters. Specifically, we used the function KMeans from SciKit-Learn^63^ with 100 initializations and two clusters. We then calculated the mean of the two cluster centers to determine the boundary between the two peaks. This process was repeated for each speech feature for later peak amplitude and latency analysis. Eventually, the boundaries were used to define an “early” and a “late” time window for each speech feature. Figure S1 shows the peaks identified and the time boundaries estimated by the K-Means clustering for all response functions and time windows are reported in Table S4.

### Statistical analyses

We ran all statistical analyses reported in this study in R (version 4.3.2). To run the LMM described in this section, we used the lme4 package^64^ for statistical model fitting and reported the $\beta$ estimates with $95\%$ confidence intervals (CI) calculated through $5\,000$ bootstrap resamples. The reports included CI, their lower and upper limits, *t*-values, and *p*-values. We normalized the continuous predictors (PTA) prior to model fitting, so the coefficients for these predictors are standardized $\beta$ coefficients. For the categorical predictors (mTRF models), we used sum contrast coding. These coefficients represent deviations from the overall mean and are not standardized in the same way as the continuous predictors.

#### Effect of MoCA group and PTA on encoding accuracy

We used a LMM to examine the encoding accuracies across mTRF models, MoCA groups, PTA, and their interactions. The LMM model was formulated as:

1 $$\begin{aligned} \text{Encoding accuracy} \sim \text{MoCA group} \times \text {PTA} {\times} \text{mTRF model} + (1 \ | \ \text {participant ID}) \end{aligned}$$

In this model, encoding accuracy, averaged across all electrodes per participant and mTRF model, served as the dependent variable. MoCA group was coded as a binary factor (0: normal, 1: low), PTA values were normalized using *z*-transformation, and the mTRF model variable was classified into five levels (acoustic, word-level segmentation, phoneme-level segmentation, word-level linguistic, and phoneme-level linguistic). We included the interaction between MoCA group and PTA to examine the effect of cognitive group on encoding accuracy while controlling for hearing ability. Additionally, we included the interaction between MoCA group and mTRF model to investigate the effect of cognitive group on encoding accuracy across different models. To examine the effect of hearing ability on encoding accuracy across different models, we included the interaction between PTA and mTRF model. Furthermore, we incorporated the three-way interaction between MoCA group, PTA, and mTRF model to investigate the combined effect of cognitive group and hearing ability on encoding accuracy across different models. By including this three-way interaction term, we aimed to assess how cognitive decline influences speech processing at various levels. We modified the default contrast coding scheme from treatment contrasts to an orthogonal sum-to-zero coding system to account for potential interactions^65^. This adjustment allowed us to estimate the main effects at the level of the grand mean, ensuring more accurate interpretation of these effects. The model also included a random intercept for the factor participant ID to account for the nested data structure.

#### mTRF-model based effects of MoCA group and PTA on response function signal power

In addition to encoding accuracy, we examined the signal power of the response functions for each mTRF model. We calculated the signal power using the RMS over delays from 0 to 500 ms for each electrode and each speech feature-based response function separately. The RMS value was chosen for two reasons: First, because of its efficiency in quantifying the total energy of the response function. RMS is a reliable measure that is robust to signal fluctuations and sensitive to the magnitude and consistency of neuronal responses by capturing potential peaks regardless of their polarity. Second, while acoustic encoding typically exhibits a P1–N1–P2 pattern, mTRF response functions in neuronal responses are less well-defined. For example, previous studies^21,49^ have identified a response to word surprisals similar to the N400 effect, characterized by a negative deflection around 400 ms after the stimulus. Using the RMS within the 0–500 ms window therefore allows us to comprehensively measure the total energy of the response function regardless of its specific shape.

A LMM with the following formula was used to examine the RMS values across mTRF models, MoCA groups, and their interaction with PTA:

2 $$\begin{aligned} \text {RMS} \sim \text {MoCA group} \times \text {PTA} + \text {speech feature} + \text {clusters} + (1 \ | \ \text {participant ID}) \end{aligned}$$

We implemented the LMM separately for each of the mTRF models. RMS was the dependent continuous variable. Again, MoCA group was coded as a binary factor (0: normal, 1: low) and PTA values were normalized using *z*-transformation. Cluster was a factor with three levels (F, C, P, with F as reference). In line with the previous statistical model, we included the interaction between MoCA group and PTA and a random intercept for the factor participant ID to account for the nested structure of the data. In the acoustic, linguistic word- and phoneme-level models, speech feature was a factor with two levels, thus two speech features per mTRF model, with the first level serving as reference level, i.e., envelope for the acoustic model, word surprisal for the linguistic word-level model, and phoneme surprisal for the linguistic phoneme-level model. Again, we used the sum-to-zero coding system to estimate the main effects at the level of the grand mean.

For the word-level and phoneme-level segmentation models, we used a slightly different formula:

3 $$\begin{aligned} \text {RMS} \sim \text {MoCA group} \times \text {PTA} + \text {clusters} + (1 \ | \ \text {participant ID}) \end{aligned}$$

In these models, only one speech feature was included, i.e., word onsets for the word-level model and phoneme onsets for the phoneme-level model, thus, there was no factor of speech feature.

We conducted post-hoc comparisons to examine the interaction between PTA and the mTRF model at word-level segmentation on the encoding accuracy using the emmeans package, with the significance level adjusted using Tukey’s method.

#### Group comparisons of peak amplitudes and latencies

Table S4 shows the percentage frequency of occurrence of peaks in three electrode clusters during early and late time windows. To examine group differences in these peak amplitudes and latencies, we focused on windows and clusters in which peaks occurred in at least $75\%$ of participants (an arbitrary threshold). Consequently, our analyses included peaks in the early window for the speech features envelope, envelope onset, word onset, and word surprisal responses, and in the late window for word onset, phoneme onset, and word surprisal responses, with different clusters for each speech feature-based response. We used two-tailed Mann–Whitney U tests to assess the differences in peak amplitudes and latencies between the normal and low MoCA groups in these time windows. Since we performed multiple comparisons over the same peaks within different clusters, we applied the Holm-Bonferroni correction^66^ to the *p*-values. This method involves adjusting the significance level of each test based on its rank among the other tests within the same response, thereby controlling the familywise error rate. Our report includes the *U*-statistic, corrected *p*-value, and effect size *r* for each comparison.

### Supplementary Information

Supplementary Information.

## Data Availability

The data used in this study are available upon request from the corresponding author.

## Code availability

The code for signal processing, including the extraction of acoustic and linguistic speech features, the Boosting pipeline, and the statistical analysis described in this paper, are available at github.com/elbolt/acuLin-speech.
