# Supplementary material for: Neural encoding of linguistic speech cues is unaffected by cognitive decline, but decreases with increasing hearing impairment
Source: Sci Rep. 2024 Aug 17;14:19105. doi: 10.1038/s41598-024-69602-1 (PMC11330478; doi:10.1038/s41598-024-69602-1)
Supplement: Supplementary file 1 — Supplementary Information. [file 41598_2024_69602_MOESM1_ESM.pdf]

Neural encoding of linguistic speech cues is unaffected by cognitive decline, but decreases with increasing hearing impairment

Supplementary information

Elena Bolt and Nathalie Giroud

August 7, 2024

Supplementary analyses

Supplementary analysis 1: MoCA score as continuous variable on encoding accuracy

In this supplementary analysis, we repeated the linear mixed model (LMM) analysis, treating the Montreal Cognitive Assessment (MoCA) score as a continuous variable. This approach contrasts with the main analysis, where participants were categorized into normal vs. low MoCA groups based on a clinical cut-off score of 26 points. The LMM used the following formula, which is identical to the one used in the main analysis, except that we treated MoCA score (normalized using z-transformation) as a continuous variable:

$$\text{Encoding accuracy} \sim \text{MoCA score} \times \text{PTA} \times \text{mTRF model} + (1 \mid \text{participant ID}) \quad (1)$$

The results are shown in Table S1. In general, this statistical model yielded the same conclusion as the one used in the main analysis. However, there was one difference, which was a significant three-way interaction between MoCA score, PTA, and the mTRF model for the linguistic word-level model.

Post-hoc tests to compare estimated marginal means across MoCA z values of -1, 0, and 1 and PTA z values of -1, 0, and 1, revealed that none of the comparisons had p-values below the typical significance threshold (i.e., $p < 0.05$), indicating that the differences in estimated marginal means (EMMs) for these conditions were not statistically significant. Specifically, for the interaction between MoCA score, PTA, and the linguistic word-level mTRF model, the estimated difference in encoding accuracy between MoCA $z = -1$ and MoCA $z = 0$ at PTA $z = -1$ was 0.004 ($SE = 0.004$, $t(50.5) = 1.020$, $p = 0.982$). The difference between MoCA $z = -1$ and MoCA $z = 1$ at PTA $z = -1$ was 0.009 ($SE = 0.009$, $t(50.5) = 1.020$, $p = 0.982$). At PTA $z = 0$, the comparison between MoCA $z = -1$ and MoCA $z = 0$ resulted in an estimated difference of 0.001 ($SE = 0.004$, $t(50.5) = 0.146$, $p = 1.000$). At PTA $z = 1$, the difference between MoCA $z = -1$ and MoCA $z = 0$ was 0.001 ($SE = 0.003$, $t(50.5) = 0.418$, $p = 1.000$).

Overall, while we observed an interaction between MoCA score, PTA, and the linguistic word-level mTRF model, post-hoc tests indicated that the differences in EMMs across these conditions were not statistically significant, suggesting that the interaction effect may not be substantial.

Supplementary analysis 2: MoCA score as continuous variable on response signal power

In line with the first supplementary analysis, we repeated the LMM analysis treating MoCA score as a continuous variable to investigate the effect of cognitive decline on the signal power of the response func-

Coefficient	β	95% CI *LL*	*UL*	df	t	p	
Intercept	0.033	0.028	0.038	40	13.0	< 0.001	***
MoCA (z)	-0.003	-0.008	0.003	40	-0.9	0.351	
PTA (z)	-0.001	-0.006	0.004	40	-0.3	0.732	
mTRF model (Seg. word-level)	0.018	0.016	0.020	160	20.0	< 0.001	***
mTRF model (Seg. phoneme-level)	-0.006	-0.008	-0.004	160	-6.3	< 0.001	***
mTRF model (Lin. word-level)	0.003	0.001	0.005	160	3.2	0.002	**
mTRF model (Lin. phoneme-level)	-0.008	-0.010	-0.006	160	-9.0	< 0.001	***
MoCA × PTA	0.004	$-1.3e-04$	0.007	40	1.9	0.065	
MoCA × mTRF model (Seg. word-level)	-0.001	-0.003	0.001	160	-0.7	0.478	
MoCA × mTRF model (Seg. phoneme-level)	0.002	$-3.7e-04$	0.004	160	1.6	0.114	
MoCA × mTRF model (Lin. word-level)	-0.002	-0.004	$3.1e-04$	160	-1.7	0.100	
MoCA × mTRF model (Lin. phoneme-level)	0.001	-0.001	0.003	160	1.4	0.161	
PTA × mTRF model (Seg. word-level)	0.004	0.002	0.005	160	3.9	< 0.001	***
PTA × mTRF model (Seg. phoneme-level)	-0.001	-0.003	0.001	160	-1.0	0.312	
PTA × mTRF model (Lin. word-level)	$-1.5e-04$	-0.002	0.002	160	-0.2	0.867	
PTA × mTRF model (Lin. phoneme-level)	-0.001	-0.003	0.001	160	-1.0	0.314	
MoCA × PTA × mTRF model (Seg. word-level)	$4.3e-04$	-0.001	0.002	160	0.6	0.528	
MoCA × PTA × mTRF model (Seg. phoneme-level)	-0.001	-0.002	$2.3e-04$	160	-1.6	0.108	
MoCA × PTA × mTRF model (Lin. word-level)	0.002	$2.6e-04$	0.003	160	2.3	0.020	*
MoCA × PTA × mTRF model (Lin. phoneme-level)	-0.001	-0.002	0.001	160	-0.7	0.458	

Table S1: Results of the linear mixed model (LMM) for the encoding accuracy of each multivariate temporal response function (mTRF) model. The LMM included Montreal Cognitive Assessment (MoCA) score, four-frequency pure-tone average (PTA), mTRF model and the interaction between MoCA, PTA and mTRF model as fixed effects, and participant ID as a random effect. The reference level for mTRF model was the acoustic model, respectively. Orthogonal contrasts were used to test the interaction effects. Significance levels are indicated as: $p < 0.001$ (***), $p < 0.01$ (**), $p < 0.05$ (*). Seg., segmentation; Lin., linguistic; CI, confidence interval; LL, lower limit; UL, upper limit; df, degrees of freedom.

tions. We quantified the signal power through the root mean square (RMS) of the response signal, which we derived from three electrodes (F, frontal; C, central; P, parietal). The LMM for the three models with two response functions nested (i.e., the acoustic and word- and phoneme-level linguistic models) took the following formula:

$$\text{RMS} \sim \text{MoCA score} \times \text{PTA} + \text{speech feature} + \text{clusters} + (1 \mid \text{participant ID}) \tag{2}$$

For the two models with one response function (i.e., the word- and phoneme-level segmentation models), the LMM took the following formula:

$$\text{RMS} \sim \text{MoCA score} \times \text{PTA} + \text{clusters} + (1 \mid \text{participant ID}) \tag{3}$$

The results are shown in Table S2. In line with the main analysis, the LMM analysis came to a comparable conclusion as the one used in the main analysis when treating MoCA score as a categorical variable. Thus, treating MoCA score as a continuous variable did not change the interpretation of the results.

Supplementary tables and figures

This document contains the following supplementary tables without additional commentary:

- Supplementary Table S3: Post-hoc tests for the main effect of the mTRF model in the main analysis.
- Supplementary Table S4: Early vs. late time windows with percentages of peak occurrence in the three electrode clusters.
- Supplementary Table S5: Peak latencies for the early and late time windows.
- Supplementary Figure S1: Time window determination through K-means clustering.

Coefficient	β	95% CI		*df*	t	p	
		LL	*UL*				
**Acoustic model**							
Intercept	0.001	0.001	0.001	57.6	15.7	< 0.001	***
MoCA (z)	$-6.0e-05$	$-2.1e-04$	$9.0e-05$	40.0	-0.8	0.437	
PTA (z)	$7.2e-05$	$-6.4e-05$	$2.1e-04$	40.0	1.0	0.306	
Speech feature (envelope onsets)	$8.6e-06$	$-6.1e-05$	$7.8e-05$	745.0	0.2	0.809	
Cluster (C)	$-6.5e-05$	$-1.5e-04$	$2.0e-05$	745.0	-1.5	0.135	
Cluster (P)	-0.001	-0.001	$-4.7e-04$	745.0	-12.8	< 0.001	***
MoCA $\times$ PTA	$5.2e-05$	$-4.9e-05$	$1.5e-04$	40.0	1.0	0.317	
**Segmentation word-level model**							
Intercept	0.001	0.001	0.001	46.4	14.8	< 0.001	***
MoCA (z)	$-4.5e-05$	$-1.9e-04$	$1.0e-04$	40.0	-0.6	0.546	
PTA (z)	$-4.7e-05$	$-1.8e-04$	$8.5e-05$	40.0	-0.7	0.486	
Cluster (C)	$1.2e-04$	$5.7e-05$	$1.8e-04$	350.0	3.7	< 0.001	***
Cluster (P)	$-2.1e-04$	$-2.7e-04$	$-1.4e-04$	350.0	-6.4	< 0.001	***
MoCA $\times$ PTA	$5.7e-05$	$-4.1e-05$	$1.6e-04$	40.0	1.1	0.258	
**Segmentation phoneme-level model**							
Intercept	0.001	0.001	0.002	45.8	13.8	< 0.001	***
MoCA (z)	$-1.4e-04$	$-3.5e-04$	$6.3e-05$	40.0	-1.4	0.182	
PTA (z)	$-5.1e-05$	$-2.4e-04$	$1.4e-04$	40.0	-0.5	0.594	
Cluster (C)	$4.1e-05$	$-4.4e-05$	$1.3e-04$	350.0	0.9	0.350	
Cluster (P)	$-4.9e-04$	-0.001	$-4.0e-04$	350.0	-11.3	< 0.001	***
MoCA $\times$ PTA	$1.1e-04$	$-3.1e-05$	$2.5e-04$	40.0	1.5	0.135	
**Linguistic word-level model**							
Intercept	0.001	0.001	0.001	67.0	19.5	< 0.001	***
MoCA (z)	$-2.7e-05$	$-9.0e-05$	$3.6e-05$	40.0	-0.8	0.408	
PTA (z)	$-2.2e-05$	$-7.9e-05$	$3.5e-05$	40.0	-0.8	0.457	
Speech feature (word frequency)	$-2.3e-04$	$-2.6e-04$	$-1.9e-04$	745.0	-12.5	< 0.001	***
Cluster (C)	$4.6e-05$	$2.0e-06$	$8.9e-05$	745.0	2.0	0.041	*
Cluster (P)	$-9.2e-05$	$-1.4e-04$	$-4.9e-05$	745.0	-4.1	< 0.001	***
MoCA $\times$ PTA	$3.0e-05$	$-1.3e-05$	$7.2e-05$	40.0	1.4	0.179	
**Linguistic phoneme-level**							
Intercept	0.001	0.001	0.001	52.9	15.6	< 0.001	***
MoCA (z)	$-5.4e-05$	$-1.4e-04$	$3.1e-05$	40.0	-1.2	0.223	
PTA (z)	$-2.9e-05$	$-1.1e-04$	$4.8e-05$	40.0	-0.7	0.462	
Speech feature (phoneme entropy)	$-1.5e-04$	$-1.8e-04$	$-1.1e-04$	745.0	-8.3	< 0.001	***
Cluster (C)	$5.2e-05$	$9.8e-06$	$9.4e-05$	745.0	2.4	0.016	*
Cluster (P)	$-1.5e-04$	$-2.0e-04$	$-1.1e-04$	745.0	-7.2	< 0.001	***
MoCA $\times$ PTA	$3.4e-05$	$-2.3e-05$	$9.2e-05$	40.0	1.2	0.248	

Table S2: Results of the linear mixed models (LMM) for the root mean square (RMS) of the temporal response function (TRF) patterns. The models included Montreal Cognitive Assessment (MoCA) score, four-frequency pure-tone average (PTA), speech feature (in the acoustic and word- and phoneme-level linguistic models), electrode cluster (F, frontal; C, central; P, parietal), and the interaction between MoCA and PTA as fixed effects, and participant ID as a random effect. The reference level for cluster was F. The reference levels for the mTRF models with two nested speech features were envelope (acoustic model), word surprise (Linguistic word-level model) and phoneme surprise (Linguistic phoneme-level model). Significance levels are indicated as: $p < 0.001$ (***), $p < 0.01$ (**), $p < 0.05$ (*). CI, confidence interval; LL, lower limit; UL, upper limit, df, degrees of freedom.

Comparison	*Estimate (EMM)*	*SE*	t	p	
Acoustic – Seg. word-level	0.024	0.0014	16.5	< 0.001	***
Acoustic – Seg. phoneme-level	0.015	0.0014	10.6	< 0.001	***
Acoustic – Lin. word-level	0.026	0.0014	18.2	< 0.001	***
Acoustic – Lin. phoneme-level	0.025	0.0014	17.5	< 0.001	***
Seg. word-level – Seg. phoneme-level	-0.008	0.0014	-5.9	< 0.001	***
Seg. word-level – Lin. word-level	0.002	0.0014	1.7	0.451	
Seg. word-level – Lin. phoneme-level	0.001	0.0014	0.9	0.882	
Seg. phoneme-level – Lin. word-level	0.011	0.0014	7.6	< 0.001	***
Seg. phoneme-level – Lin. phoneme-level	0.010	0.0014	6.8	< 0.001	***
Lin. word-level - Lin. phoneme-level	-0.001	0.0014	-0.7	0.947	

Table S3: Post-hoc tests for the main effect of the multivariate temporal response function (mTRF) model. The table shows the estimated differences in the estimated marginal means (EMMs), standard errors (SE), t-, and p-values for pairwise comparisons between the acoustic model and the other models, as well as among the segmentation and linguistic models. Significance levels are indicated as: $p < 0.001$ (***). Seg., segmentation; Lin., linguistic., SE, standard error.

		Percentage in clusters		
Speech feature	Time range	F	C	P
Early window				
Envelope	0–244 ms	97.7^+	93.2^+	86.4^+
Envelope onsets	0–90 ms	81.8^+	68.2	50.0
Word onset	0–169 ms	88.6^+	93.2^+	77.3^+
Phoneme onset	0–139 ms	100.0^+	95.5^+	84.1^+
Word surprisal	0–129 ms	75.0^+	90.9^+	79.5^+
Word frequency	0–181 ms	68.2	68.2	59.1
Phoneme surprisal	0–157 ms	81.8^+	84.1^+	63.6
Phoneme entropy	0–158 ms	84.1^+	84.1^+	81.8^+
Late window				
Envelope	245–500 ms	15.9	22.7	43.2
Envelope onsets	91–500 ms	65.9	56.8	56.8
Word onset	170–500 ms	88.6^+	81.8^+	84.1^+
Phoneme onset	140–500 ms	65.9	56.8	72.7
Word surprisal	130–500 ms	90.9^+	75.0^+	77.3^+
Word frequency	182–500 ms	79.5^+	68.2	75.0^+
Phoneme surprisal	158–500 ms	68.2	40.9	59.1
Phoneme entropy	159–500 ms	65.9	61.4	70.5

Table S4: Percentage of occurrence of peaks in the three electrode clusters of interest and time boundaries for the early and late time windows. The $^+$ indicates that the peak occurred in more than 75% of participants. F, frontal; C, central; P, parietal.

Speech feature	Cluster	Peak latencies by MoCA group					
		Normal			Low		
		n	*M*	*SD*	*n*	*M*	*SD*
Early window							
Envelope	F	25	124.8	30.6	18	130.9	36.6
	C	23	123.0	28.1	18	124.2	33.1
	P	22	124.4	40.6	17	114.1	46.6
Envelope onsets	F	25	8.1	11.5	18	7.0	11.1
Word onset	F	22	58.9	15.3	17	59.2	16.4
	C	23	56.5	15.0	18	54.8	17.0
	P	18	50.2	28.8	17	53.4	17.2
Phoneme onset	F	25	21.9	9.0	19	22.0	6.5
	C	25	21.9	11.3	18	23.1	7.2
	P	22	43.3	40.4	16	16.8	11.9
Word surprisal	F	18	58.2	17.5	15	56.2	11.9
	C	22	59.2	13.8	18	57.4	23.7
	P	19	58.2	35.1	16	48.0	19.5
Phoneme surprisal	F	21	30.5	30.9	15	25.3	20.5
	C	23	36.6	40.3	15	27.1	20.8
Phoneme entropy	F	23	21.5	16.8	16	23.6	8.0
	C	22	21.6	14.6	17	20.6	10.9
	P	22	48.0	41.3	16	27.5	35.6
Late window							
Word onset	F	22	270.2	43.6	17	256.8	42.6
	C	20	246.1	50.1	16	246.3	37.4
	P	22	248.5	55.5	15	271.4	75.7
Word surprisal	F	22	266.8	45.4	18	255.7	51.1
	C	18	237.5	39.3	15	251.6	36.2
	P	19	247.4	69.4	15	272.7	67.2
Word frequency	F	20	323.0	50.4	15	306.5	30.2
	P	18	301.5	50.1	15	284.1	68.1

Table S5: Descriptive statistics of peak latencies for each response to speech feature in the early and late time windows by electrode cluster of interest, separated by Montreal Cognitive Assessment (MoCA) group. Note that the table only shows the peaks that occurred in more than 75% of the participants. M, mean; SD, standard deviation; F, frontal; C, central; P, parietal.

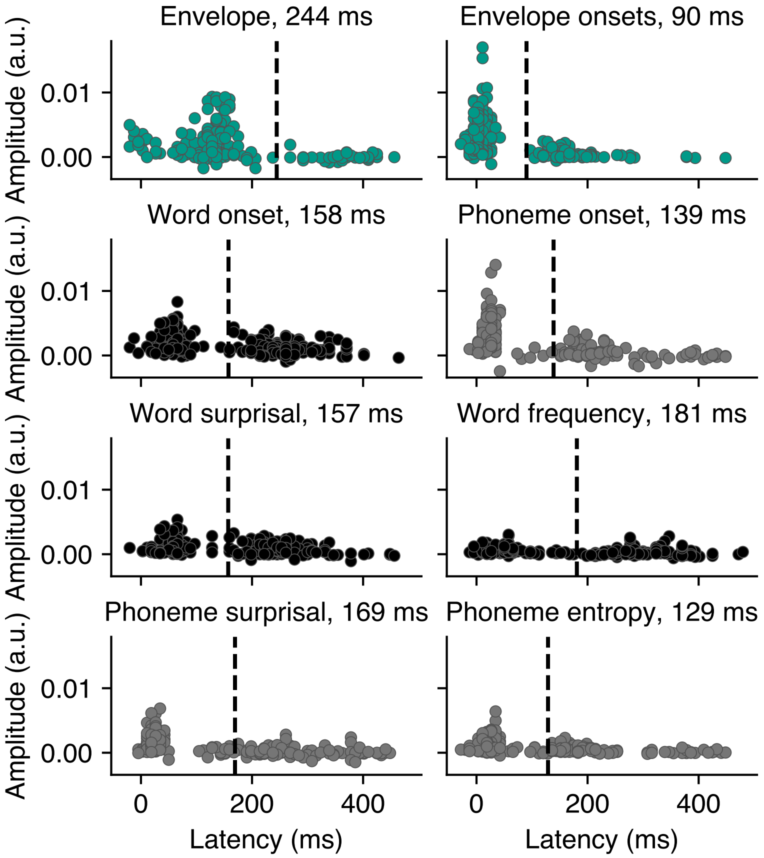

Figure S1: Response peaks for each speech feature-based response function, identified in the positive time lags of three midline clusters (F, frontal; C, central; P, parietal). The dashed line indicates the decision boundaries determined by the K-Means clustering algorithm, which we used to identify early and late time windows in group comparisons.

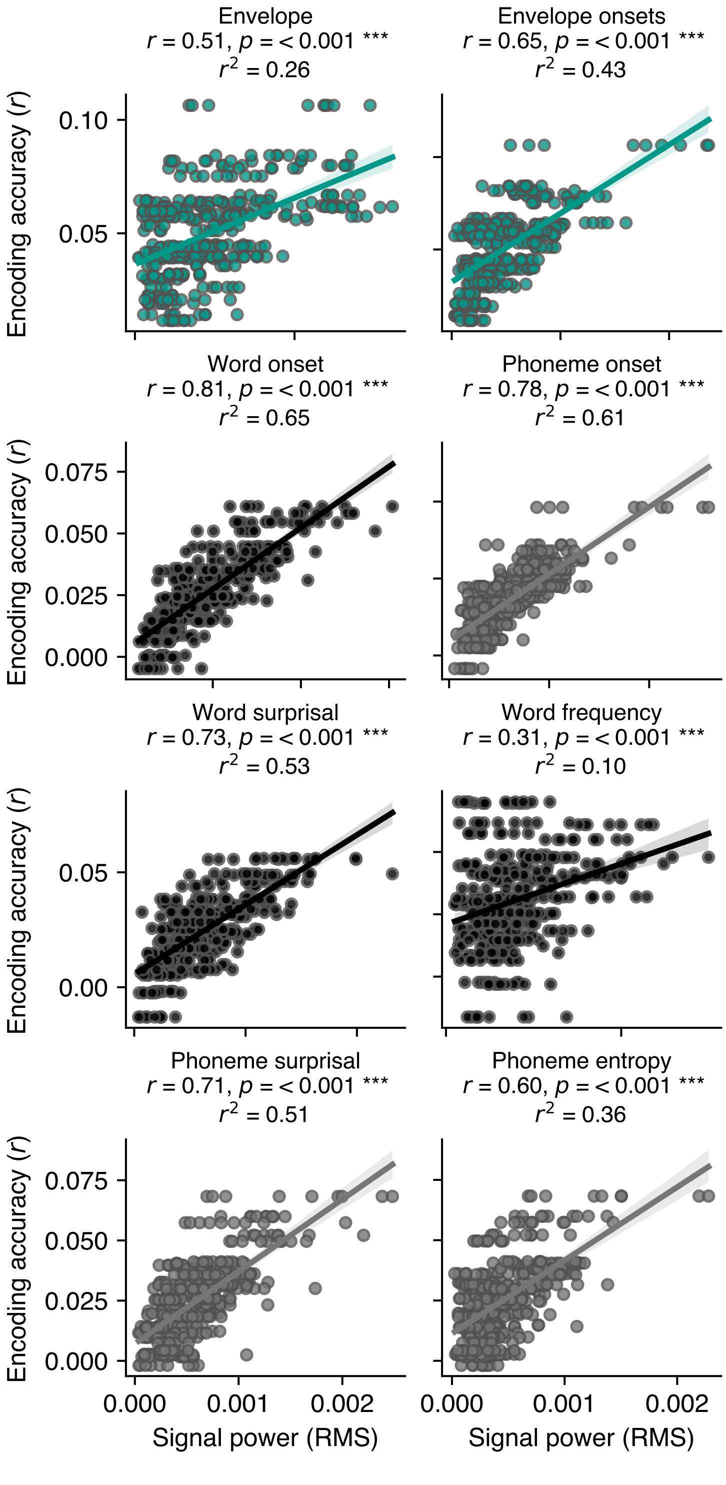

Figure S2: Scatterplots with regression lines showing the association between mTRF model encoding accuracy score (r) and the root mean square (RMS) of the response signal power for each speech-feature based response. The plots illustrate the linear relationship between RMS and the score they were nested in, for the acoustic model, segmentation word- and phoneme-level models, and linguistic word- and phoneme-level models, respectively. All Pearson correlation coefficients are significant, with significance levels indicated as $p < 0.001$ (***). The r^2 values are also provided for each plot, showing the proportion of variance explained by the linear relationship. The shaded area around the regression line represents the 95% confidence interval.
